# Adjustment and maladjustment to later life: Evidence about early experiences in the family

**DOI:** 10.3389/fpsyg.2023.1059458

**Published:** 2023-03-02

**Authors:** Marta Alcaide, Oscar F. Garcia, Pablo Queiroz, Fernando Garcia

**Affiliations:** ^1^Department of Methodology of the Behavioural Sciences, Faculty of Psychology, University of Valencia, Valencia, Spain; ^2^Department of Developmental and Educational Psychology, University of Valencia, Valencia, Spain; ^3^Faculty of Health Sciences, Federal University of Rio Grande do Norte, Trairi, Brazil

**Keywords:** parenting styles, warmth, strictness, life cycle, later life

## Abstract

**Introduction:**

Previous parenting studies with European-American families have identified optimal parenting as being based on warmth combined with strictness (i.e., authoritative parenting). The present study analyzes, in adolescents and adults, their adjustment and maladjustment related to age and their early experiences in the family.

**Methods:**

The sample consisted of 2,158 Spanish people (58.29% women): 624 adolescents, 630 young adults, 504 middle-aged adults, and 400 older adults. The families were classified into one of the four parental typologies (authoritative, indulgent, authoritarian, and neglectful) based on their scores in the two main dimensions (warmth and strictness). Child functioning was measured as components of adjustment (self-esteem, emotional self-concept, and empathy) and maladjustment (aggression and hostile sexism).

**Results:**

Overall, only adolescents and adult children raised in indulgent families reported the highest self-esteem, emotional self-concept, and empathy, and the lowest aggression and hostile sexism. Authoritative parenting (warmth with strictness) was related to a lower emotional self-concept and greater aggression and hostile sexism than indulgent parenting (warmth without strictness). The worst scores corresponded to authoritarian and neglectful parenting.

**Discussion:**

The present findings provide new evidence about early experiences in the family, even after parental socialization has ended. Interestingly, contrary to the main findings from classic studies with European-American families, only high parental warmth combined with low parental strictness (i.e., indulgent parenting) is always positive for greater adjustment and less maladjustment in all age groups.

## 1. Introduction

At any moment in life, the different components of adjustment and maladjustment can be related to age (Baltes, 1987). An old question is how these personal differences are affected by early experiences in the family, e.g., old psychoanalytic paradigm (Maccoby, 1992; Darling and Steinberg, 1993; Garcia et al., 2019). From a cross-sectional 21st-century perspective where individuals of all ages live side by side, the present study analyzes participants’ adjustment and maladjustment related to their age and their early experiences in the family, taking into account the parenting style.

### 1.1. Age-related differences in adjustment and maladjustment

From a developmental perspective, adjustment and maladjustment can vary depending on the phase of the life span (Mcadams et al., 1993; Liu et al., 2019; Buchinger et al., 2021). Later life is characterized by a loss of primary control capacity and decreasing opportunities associated with reductions in personal and social resources (Baltes, 1987), such as increases in health problems (e.g., cognitive decline) and fewer social roles (e.g., retirement). By contrast, adulthood is usually related to several developmental tasks that contribute to successful development from young adulthood (e.g., university studies) to midlife (e.g., progression of career), when middle-adults achieve high levels of achievement, mastery, and control over the self and the environment (Liu et al., 2019; Buchinger et al., 2021).

Consequently, there is an age-related decline in the self in old age (Orth et al., 2010, 2018). Overall, older adults have a poorer sense of their own worth (i.e., self-esteem) in comparison with middle-life (the peak levels) and young adulthood. Even in later life, scores on self-esteem might be similar to those of adolescence (Robins and Trzesniewski, 2005; Liu et al., 2019), and so older adults might be vulnerable to problems such as depressive symptoms and low self-rated health, less ego integrity, and more despair and death anxiety (Bergman and Bodner, 2020). Nevertheless, despite the pattern of greater losses than gains, adaptive changes appear in older life; specifically, emotion regulation seems to be relatively spared (Carstensen and Mikels, 2005). Based on a multidimensional approach to the self (i.e., self-concept), there might be an age-related decline in the emotional component of the self (self-concept), as has been described in self-esteem (Liu et al., 2019), but it can also be preserved due to relatively good emotion regulation in later life (Carstensen and Mikels, 2005). However, this point has not been exhaustively tested. Additionally, empathy (i.e., concern for the other) is an important component of adjustment throughout life, and it is positively related to prosocial behaviors and is a protective factor against deviance (Eisenberg and Strayer, 1987). Empathy seems to decrease with age, particularly related to understanding more complex emotions and mental states (Henry et al., 2013). However, there is limited evidence, and it is mostly based on comparisons of young and older adults (e.g., Bailey et al., 2008; Henry et al., 2013).

By contrast, aggression and prejudice are commonly identified as important components of maladjustment and poor functioning (Donnellan et al., 2005; Gracia et al., 2008). Aggressors are more likely to show antisocial behavior, conduct problems, lack of self-control and empathy, and an increased risk of internalizing problems, such as anxiety and depression (Donnellan et al., 2005). Overall, aggression is related to greater maladjustment in later life, but also in adulthood (Liu et al., 2013) and adolescence (Perez-Gramaje et al., 2020). Additionally, higher scores on prejudice are also related to more aggression and discrimination (Genthner and Taylor, 1973; Gonsalkorale et al., 2009), and it seems to be greater in later life (Hammond et al., 2018). Particularly, prejudice against women (e.g., hostile sexism) has been studied less in later life (Hammond et al., 2018), given that most of the studies have been limited to more classic forms such as racial prejudice (e.g., Gonsalkorale et al., 2009).

Although previous studies have examined some variations in their functioning in older adults, adjustment and maladjustment in later life, compared to adulthood and adolescence, are not clearly identified. They could reflect age-related declines in some domains (e.g., self-esteem, Liu et al., 2019), whereas others seem to be preserved (e.g., emotion regulation, Carstensen and Mikels, 2005). Determining age-related differences in different dimensions of adjustment and maladjustment is important for identifying moments of vulnerability or strength throughout the life cycle. For example, adolescents and older adults tend to report lower self-esteem than middle-aged adults, making them more likely to have certain problems and difficulties. Much of the research has been limited to comparing a few groups (e.g., young adults and older adults), or it has considered only a few similar variables (e.g., empathy, Bailey et al., 2008), but not broad and different dimensions of adjustment and maladjustment at the same time.

### 1.2. Early experiences in the family and their relationship with adjustment and maladjustment

The pattern of adjustment and maladjustment during later life and adulthood could be especially related to early experiences (e.g., peers and family), as many developmental scholars have proposed (Freud, 1933; Baumrind, 1978; Maccoby, 1992; Darling and Steinberg, 1993). Early experiences in the family during parental socialization are crucial for children and adolescents when they are being raised by their parents, but they could also have a long-term impact beyond adolescence. Parents help the child to become an adult, but there is a time when parental socialization is over and the child reaches adulthood (Stafford et al., 2015, 2016). At any time in life, there are differences between individuals with higher well-being and less maladjustment and those with poor adjustment and greater problems. These differences are already described in childhood (Eisenberg et al., 1998; Ridao et al., 2021; Hung, 2022), but they become greater in adolescence, for example, between adolescents with high and low academic engagement (Veiga et al., 2021), empathy (Musitu-Ferrer et al., 2019), and self-concept (Veiga et al., 2015), perhaps partly because adolescents spend more time with their peers without adult supervision and are more susceptible to group standards. The same is true of differences in adjustment and maladjustment among adults (Scales et al., 2016; Orth et al., 2018). The family influences the development of children (Gomez-Ortiz and Sanchez-Sanchez, 2022; Sandoval-Obando et al., 2022), and, particularly, the so-called parental socialization can be consistently related to differences in child adjustment and maladjustment during childhood (Baumrind, 1967; Baumrind, 1971) and adolescence (Lamborn et al., 1991; Steinberg et al., 1992; Garcia and Gracia, 2009). Additionally, less is known about whether differences in adjustment and maladjustment during adulthood can be consistently related to parental socialization.

Many classic and current studies on parental socialization are based on the model with two independent dimensions (i.e., orthogonal) whose combination gives rise to four parenting styles (Maccoby and Martin, 1983; Darling and Steinberg, 1993; Martínez et al., 2017; Garcia et al., 2019). The warmth dimension (also called responsiveness, security, care, love, or acceptance/involvement) refers to the degree to which parents show acceptance, emotional closeness, and communication with their children (Lamborn et al., 1991; Martinez-Escudero et al., 2020; Martínez et al., 2021). The strictness dimension (also known as demandingness, severity, or supervision) refers to the degree of imposition and rigidity with which parents act to establish rules, imposing their authority (Darling and Steinberg, 1993; Martinez et al., 2019; Garcia et al., 2020a,b). Four parenting styles emerge from the combination of the two orthogonal dimensions: authoritative (high warmth and high strictness), indulgent (high warmth and low strictness), authoritarian (low warmth and high strictness), and neglectful (low warmth and low strictness).

Parents transmit social norms and values to make their children autonomous, mature, and independent individuals (Darling and Steinberg, 1993; Climent-Galarza et al., 2022). Parental socialization takes place in very different social, cultural, and ethnic contexts (Deater-Deckard et al., 1996; Chao, 2001; Dwairy et al., 2006; Fuentes et al., 2022). For example, poor neighborhoods tend to have fewer opportunities, higher unemployment, worse schools, and greater crime rates than middle-class neighborhoods (Steinberg et al., 2006; Sandoval-Obando et al., 2022). However, parental socialization can be consistently related to differences in adjustment, so it is possible to identify parenting styles that are positive for children’s well-being and parenting styles that are related to detrimental consequences (Lamborn et al., 1991; Fuentes et al., 2022). In parental socialization research, for each specific context, families can be characterized in terms of high or low levels of warmth and strictness. For example, authoritarian families are less warm and stricter than other families, but the characterization of each family based on warmth and strictness is sample specific, depending on the ethnicity, e.g., European-American (Baumrind, 1967) and African-American (Baumrind, 1972), or country, e.g., Sweden, United Kingdom, Portugal or Slovenia (Calafat et al., 2014). The key issue is that there may be variations in the optimal parenting style depending on the culture (Pinquart and Kauser, 2018), and the authoritative style (i.e., strictness combined with warmth) may not be universally beneficial in all cultural contexts (Palacios et al., 2022).

Authoritative parenting (i.e., warmth and strictness) has been related to optimal levels of psychosocial adjustment mostly in studies in the Anglo-Saxon context with European-American families (Baumrind, 1967; Baumrind, 1971; Maccoby and Martin, 1983; Darling and Steinberg, 1993; Steinberg et al., 1994; Steinberg, 2001). Benefits of authoritative parenting have been found for different criteria, such as self-esteem, mental health (Maccoby and Martin, 1983), psychosocial development, school performance, internalized distress, and behavioral problems (Lamborn et al., 1991; Steinberg et al., 1994). However, authoritative parenting is not always associated with the optimal adjustment of children and adolescents across all cultural contexts (Darling and Steinberg, 1993; Chao, 2001; Pinquart and Kauser, 2018; Garcia et al., 2019).

Some studies conducted in ethnic minorities in the United States, such as African-American (Baumrind, 1972) and Chinese-American (Chao, 2001), as well as in Arab societies (Dwairy and Achoui, 2006), report that the authoritarian parenting style (i.e., strictness without warmth) is related to some benefits in psychosocial adjustment, for example, greater assertiveness and independence in African Americans (Baumrind, 1972) and an absence of mental health problems in Arab adolescents (Dwairy and Achoui, 2006).

Furthermore, a body of recent research, mostly from studies conducted in Europe and Latin America, points to the benefits of indulgent parenting in raising children (Calafat et al., 2014; Garcia et al., 2019; Martinez et al., 2020). Indulgent parenting (i.e., warmth without strictness) is associated with equal or better psychosocial adjustment than authoritative parenting (i.e., warmth and strictness), whereas the authoritarian and neglectful parenting styles (i.e., without warmth) are associated with worse levels of adjustment (Villarejo et al., 2020; Gimenez-Serrano et al., 2021). Children from indulgent families present better adjustment than children from authoritative families on criteria such as self-esteem and environmental values (Queiroz et al., 2020), alcohol use and abuse, and motivations for drinking and non-drinking (Garcia et al., 2020b), as well as a wide spectrum of behavioral problems (Fuentes et al., 2015a).

Additionally, it is commonly conjectured that early experiences in the family (e.g., parental socialization) could be related to adjustment and maladjustment in adulthood and later life. However, little is known about the relationship between parental socialization and adult adjustment and maladjustment, given that the empirical evidence is mostly limited to examining parenting and its consequences in children and adolescents (Maccoby and Martin, 1983; Lamborn et al., 1991; Riquelme et al., 2018; Queiroz et al., 2020). A few studies have tested the long-term socialization impact of parenting beyond adolescence, but most of them have been limited to samples of young adults (Garcia et al., 2021), used different outcomes in the comparisons across adulthood (Stafford et al., 2016), included adult children but not adolescents at the same time (Stafford et al., 2015; Gimenez-Serrano et al., 2022) or compared adolescents with only one group of adults (Garcia O. F. et al., 2018), examined specific adult ages (e.g., 43 years) but not age groups (e.g., middle-aged adults; Stafford et al., 2016), or used isolated parenting practices instead of parenting styles (Ali et al., 2015).

### 1.3. The present study

The present study examined age-related differences in adjustment (self-esteem, emotional self-concept, and empathy) and maladjustment (aggression and hostile sexism) from adolescence to later life in a European country (i.e., Spain). Based on previous studies, older adults were expected to score negatively on self-esteem, empathy, and hostile sexism, but more positively on outcomes associated with good emotion regulation in old age (lower aggression and higher emotional self-concept). It is of interest to ask whether, despite the expected pattern of age-related differences, variations in adjustment and maladjustment among the four age groups (adolescence, young and middle adulthood, and later life) might also be consistently associated with early experiences in the family (authoritative, indulgent, authoritarian, and neglectful). Based on some recent studies, we hypothesized that indulgent parenting would be related to equal and even more positive scores than authoritative parenting in terms of greater adjustment (higher self-esteem, emotional self-concept, and empathy) and less maladjustment (lower aggression and hostile sexism), whereas the most negative scores would be associated with parenting characterized by lack of warmth (authoritarian and neglectful parenting styles).

## 2. Methods

### 2.1. Sample and procedure

The sample consisted of 2,158 Spanish participants, 1,258 females (58.30%) and 900 males (41.70%), who were adolescents and adult children (*M* = 35.74, SD = 20.26) from four age groups: adolescents (*n* = 624, 362 females, 58%), aged 12–18 years (*M* = 16.69, SD = 1.60); young adults (*n* = 630, 376 females, 59.7%), aged 19–35 years (*M* = 23.60, SD = 3.73); middle-aged adults (*n* = 504, 312 females, 61.9%), aged 36–59 years (*M* = 48.41, SD = 6.37); and older adults (*n* = 400, 208 females, 52%), aged 60 years or older (*M* = 68.61, SD = 7.47). Following the *a priori* power analysis (Faul et al., 2009), a minimum sample of 1,724 participants was required to detect with a statistical power of 0.95 (by fixing the conventional values of Type I and Type II statistical inference errors: *α* = 0.05; 1 − *β* = 0.95) and the low effect size generally identified in parenting styles, *f* = 0.10 (Cohen, 1977; Lamborn et al., 1991; Garcia et al., 2008; Faul et al., 2009). For instance, a small effect size (*f* = 0.17) was estimated from ANOVAs from a classic parenting study based on univariate *F*-tests of the four parenting styles and a large number of dependent variables (Lamborn et al., 1991). However, the effect size of parenting styles depends on the criterion measured (e.g., greater relationship with family self-concept than with general self-esteem). In this study, we expect the effect size to be small because the constructs used as criteria are especially broad. It is therefore important to ensure that the sample has sufficient power to detect these possible small sizes (see Lamborn et al., 1991, p. 1063). A sensitivity power analysis with the study sample (*N* = 2,158, *α* = *β* = 0.05) showed that main effects between the four parenting styles can detect statistically significant differences with a very small effect size (*f* = 0.089; Faul et al., 2009; Garcia and Gracia, 2009; Calafat et al., 2014; Fuentes et al., 2015b). G-power 3.1 was used to calculate the statistical power (Faul et al., 2009).

Adolescents were recruited from the complete list of high schools. The heads of all the high schools invited to participate were contacted. If a high school declined to be part of the study, an alternative school from the complete list was chosen until completing the sample size required (Garcia O. F. et al., 2018; Martínez et al., 2019). Parental consent was mandatory for adolescent participation. Young adults were recruited in undergraduate education courses (Manzeske and Stright, 2009; Garcia et al., 2021). Middle-aged adults were recruited from city council neighborhoods (Garcia et al., 2011). Older adults were recruited from the complete list of senior citizen centers. If a senior citizen center refused to be part of the study, an alternative center from the complete list was chosen until completing the sample size required (Garcia O. F. et al., 2018). Data were gathered by using an online survey with obligatory responses. To ensure data protection measures, identifiers and survey data were stored in separate archives, passwords to directories were protected, and sensitive files were encoded. The questionnaires were studied for doubtful response patterns, such as describing implausible inconsistencies between negatively and positively formulated responses (Tomás and Oliver, 2004; Garcia et al., 2011). Subjects who participated in the following study met the following requirements: (1) They were Spanish, as were their parents and grandparents; (2) Participation was voluntary; (3) Informed consent was required; and (4) Anonymity of responses was guaranteed.

### 2.2. Measures

#### 2.2.1. Parental socialization

The warmth dimension was measured with the 20 items on the Warmth/Affection Scale (Rohner et al., 1978), which assesses the degree to which children perceive their parents to be affectionate, responsive, and involved in the child’s affairs. This scale provides a reliable measure of adolescents’ perceptions of their parents’ behaviors. A sample item is “Make me feel wanted and needed.” The adult version includes the same statements in past tense, offering a reliable measure for adult children (Ali et al., 2015). A sample item is “Made me feel what I did was important.” The alpha value was 0.946. The strictness dimension was measured with the 13 items on the Parental Control Scale (Rohner et al., 1978). It evaluates the degree to which children perceive that their parents control and monitor them in an imposing, firm, and demanding manner. A sample item is “Insist that I do exactly as I am told.” The adult version has the same statements in past tense, offering a reliable measure for adult children (Ali et al., 2015). A sample item is “Insisted that I do exactly as I was told.” The alpha value was 0.903. Both scales are 4-point Likert-type scales ranging from 1 *Almost never is/was true* to 4 *Almost always is/was true*. High scores on both scales indicate a higher degree of warmth and strictness. Overall, parenting studies with adults use the same measure to capture parental socialization as the one used for adolescents, but the items are written in the past tense (Buri, 1991; Khaleque and Rohner, 2002a). The Warmth/Affection Scale and the Parental Control Scale offer reliable and valid measures to capture parental socialization in children, but also in adults once parental socialization has ended (Khaleque and Rohner, 2002a,b; Rohner and Khaleque, 2003). Additionally, the factor structures for both scales have been confirmed with CFA analysis, as well as their invariance by age (Garcia O. F. et al., 2018).

The four parenting styles were defined using the median split procedure in the two parental dimensions (i.e., warmth and strictness), based on the sex and age of the participants (Chao, 2001; Calafat et al., 2014; Queiroz et al., 2020; Garcia et al., 2020a,b). In authoritative families, the scores were above the median in both parental dimensions, whereas in neglectful families, the scores were below the median in both parental dimensions. In indulgent families, the scores were above the median for parental warmth, but below it for parental strictness, whereas in authoritarian families, the scores were below the median for parental warmth, but above it for parental strictness.

The split procedure to assign families to the parenting styles is widely used in the specialized literature on parenting, with cutoff criteria based on the tertile-split procedure (Lamborn et al., 1991; Steinberg et al., 1994; Garcia and Gracia, 2009) or the median-split procedure (Chao, 2001; Calafat et al., 2014; Queiroz et al., 2020). This procedure to categorize families allows us to assess both of the main parenting dimensions with different measures, such as the APM (Authoritative Parenting Measure; Lamborn et al., 1991; Steinberg et al., 1994); WAS Warmth/Affection Scale (Rohner et al., 1978) and PCS Parental Control Scale (Rohner and Khaleque, 2003); the EMBU questionnaire (Arrindell et al., 1999); or the ESPA29 Parental Socialization Scale (Musitu and Garcia, 2001). As this procedure is sample-specific, it allows us to simultaneously divide the two main parenting dimensions into high and low scores, controlling for sociodemographic variables (e.g., sex, age, and country; Garcia and Gracia, 2009; Calafat et al., 2014). Although we can be confident that the parents in our “neglectful” category are indeed relatively more neglectful (i.e., less warm and less strict) than the other parents in the sample, we do not know whether the parents we have labeled “neglectful” would be considered “neglectful” in a different population. Therefore, it is important to consider that assigning parents to one type or another relative to their counterparts is done for heuristic, rather than diagnostic, purposes (see Lamborn et al., 1991, p. 1053).

#### 2.2.2. Emotional self-concept

It was measured with the six items on the emotional scale from the AF5 Self-Concept Form 5 (Garcia F. et al., 2018; Garcia O. F. et al., 2018; Chen et al., 2020). This scale assesses the general self-perception of the emotional state and responses to specific situations in daily life that require a certain degree of commitment and involvement (Fuentes et al., 2020). A sample item is “Many things make me nervous” (reversed item). A high score corresponds to a higher emotional self-concept. Its response scale is from 1 to 99, where 1 is *Very little agreement* and 99 is *Very much in agreement*. The alpha value was 0.762.

#### 2.2.3. Self-esteem

It was measured with the 10 items on the Rosenberg questionnaire (Rosenberg, 1965). This instrument evaluates feelings of self-worth and self-acceptance. A sample item is “I feel that I am a person of worth, at least on an equal plane with others.” It consists of a 4-point Likert-type response scale, ranging from 1 *Completely disagree* to 4 *Completely agree*. Higher scores represent higher self-esteem. The alpha value was 0.850.

#### 2.2.4. Empathy

It was measured with the five items on the empathy scale of the Psychosocial Maturity Questionnaire (CRPM3; Greenberger et al., 1975; Garcia and Serra, 2019). This scale assesses the degree to which a person can understand the point of view of the other (perceive their feelings and emotions) and elicit an emotional response congruent with the other’s emotional state and needs. A sample item is “I am sensitive to others’ feelings and needs.” It consists of a 5-point Likert-type response scale, where 1 is *Strongly disagree* and 5 is *Strongly agree*. Higher scores correspond to greater empathy. The alpha value was 0.683.

#### 2.2.5. Aggression

It was measured with the six items on the Hostility/Aggression Scale of the Personality Assessment Questionnaire (PAQ; Rohner, 1978), which assesses personality self-perception and behavioral traits linked to hostile and aggressive tendencies (Rohner, 2014; Ali et al., 2015). The Hostility/Aggression Scale is commonly used in the specialized literature on parenting and child adjustment (Khaleque and Rohner, 2002a; Lila et al., 2007; Ali et al., 2015, 2019). A sample item is “I want to hit something or someone.” It consists of a 4-point Likert-type response scale, where 1 is *Almost never true* and 4 is *Almost always true*. Higher scores on this scale indicate greater aggression. The alpha value was 0.659.

#### 2.2.6. Hostile sexism

It was measured with the 11 items on the hostile sexism scale of the Ambivalent Sexism Inventory (ASI; Glick and Fiske, 1996), which assesses the degree of prejudice against women, considering them inferior to men. A sample item is “Women are too easily offended.” It consists of a 6-point Likert-type response scale, where 1 is *Strongly Disagree* and 6 is *Strongly Agree*. Higher scores indicate greater hostile sexism. The alpha value was 0.931.

### 2.3. Plan of analysis

The statistical analyses performed were multivariate analyses (MANOVA), univariate analyses (ANOVA), and Bonferroni hypothesis tests. First, a multivariate factorial design MANOVA (4 × 2 × 4) was applied, in which the dependent variables were the indicators of adjustment (i.e., self-esteem, emotional self-concept, and empathy) and maladjustment (i.e., aggression and hostile sexism), and the independent variables were the parenting styles (authoritative, indulgent, authoritarian, and neglectful), sex (male and female), and age groups (adolescents, aged 12–18 years; young adults, aged 19–35 years; middle-aged adults, aged 40–59 years; and older adults, aged 60 years or more). Second, univariate tests were used to analyze sources of variation that reached statistical significance in the multivariate analyses. Third, *post-hoc* Bonferroni tests were applied to those univariate sources of significance while maintaining the alpha per study at 5%. The IBM SPSS 28.0.1.1 statistical program was used for the MANOVAs, ANOVAs, and differences between pairs of means, with Bonferroni correction for rate of Type I error.

## 3. Results

### 3.1. Parenting styles

The participants were distributed by parenting styles (see Table 1). The indulgent parenting (*M* = 73.60, SD = 4.51) and authoritative parenting (*M* = 72.52, SD = 4.36) styles had higher scores on warmth, compared to the authoritarian parenting (*M* = 55.22, SD = 9.98) and neglectful parenting (*M* = 56.69, SD = 9.62) styles. Additionally, authoritative parenting (*M* = 39.78, SD = 5.10) and authoritarian parenting (*M* = 41.65, SD = 5.55) showed higher scores on strictness than indulgent parenting (*M* = 28.17, SD = 5.41) and neglectful parenting (*M* = 28.07, SD = 5.77).

**Table 1:** Distribution of participants according to parenting style, mean scores (*M*), and standard deviations (SD) in the main parenting dimensions.

	Total	Indulgent	Authoritative	Authoritarian	Neglectful
Frequency	2,158	607	461	632	458
Percentage	100	28.1	21.4	29.3	21.2
Warmth
Mean	64.40	73.60	72.52	55.22	56.69
SD	11.57	4.51	4.36	9.98	9.62
Strictness
Mean	34.58	28.17	39.78	41.65	28.07
SD	8.42	5.41	5.10	5.55	5.77

### 3.2. Preliminary multivariate analyses

The results of the multivariate analyses showed statistically significant differences in the main effects of parenting style, Λ = 0.879, *F*(15.0, 5858.3) = 18.64, *p* < 0.001, sex, Λ = 0.844, *F*(5.0, 2122.0) = 78.44, *p* < 0.001, and age, Λ = 0.895, *F*(15.0, 5858.3) = 14.84, *p* < 0.001, and in the interaction effects of parenting style by age, Λ = 0.969, *F*(45.0, 9495.3) = 1.47 (see Table 2).

**Table 2:** Factorial (4^a^ × 2^b^ × 4^c^) for adjustment (self-esteem, emotional self-concept, and empathy) and maladjustment (aggression and hostile sexism) criteria.

Sources of variation	Λ	*F*	*df*_between_	*df*_error_	Value of *p*
Parenting styles^a^	0.879	18.64	15.0	5858.3	<0.001
Sex^b^	0.844	78.44	5.0	2122.0	<0.001
Age^c^	0.902	14.84	15.0	5858.3	<0.001
Parenting styles × Sex	0.995	0.72	15.0	5858.3	0.762
Parenting styles × Age	0.969	1.47	45.0	9495.3	0.022
Sex × Age	0.988	1.66	15.0	5858.3	0.052
Parenting styles × Sex × Age	0.977	1.12	45.0	9495.3	0.267

a_1_ indulgent, a_2_ authoritative, a_3_ authoritarian, a_4_ neglectful. b_1_ female, and b_2_ male. c_1_ adolescents (12–18 years), c_2_ young adults (19–35 years), c_3_ middle-aged adults (36–59 years), and c_4_ older adults (60 years and older).

### 3.3. Adjustment and maladjustment throughout life

**Figure 1:**
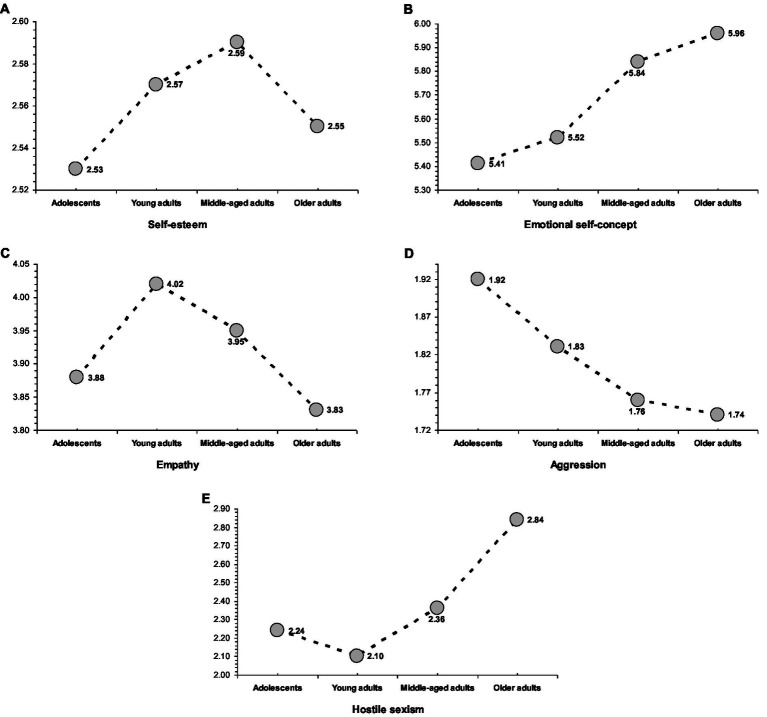
Age profiles in adjustment criteria, self-esteem **(A)**, emotional self-concept **(B)**, and empathy **(C)**, and maladjustment criteria, aggression **(D)** and hostile sexism **(E)**.

Statistically significant age differences were found in all the adjustment and maladjustment criteria, *p* < 0.01 (see Table 3). Examining the age profiles in the adjustment indicators (see Figure 1), self-esteem showed an age-related increasing tendency from adolescence (low levels), reaching a peak in middle-adulthood and then subsiding in later life. Middle-aged adults reported higher self-esteem than older adults. Moreover, adolescents reported lower self-esteem than the three adult age groups. Emotional self-concept showed an age-related increasing tendency from low levels in adolescence to high levels in later life. Specifically, scores on emotional self-concept were higher in older and middle-aged adults than in young adults and adolescents. The age profile for empathy showed that scores were low in adolescence, rising to a peak in young adulthood, and then declining in middle life and later life. The lowest empathy corresponded to older adults and adolescents (who did not differ from each other), whereas young adults were the most empathic. Additionally, middle-aged adults had higher scores than older adults.

**Table 3:** Means and (standard deviations) for age group, univariate *F*-values, and (*f* Cohen) for adjustment (self-esteem, emotional self-concept, and empathy) and maladjustment (aggression and hostile sexism) criteria.

	Age				*F*(3, 2,126)	Value of *p*
	Adolescents (12–18 years old)	Young adults (19–35 years old)	Middle-aged adults (36–59 years old)	Older adults (>60 years old)		
Adjustment
Self-esteem	2.53^b^	2.57^a^	2.59^a, 1^	2.55ª^, 2^	9.23	< 0.001
	(0.20)	(0.19)	(0.19)	(0.20)	(0.114)	
Emotional self-concept	5.41^b^	5.52^b^	5.84^a^	5.96^a^	10.07	< 0.001
	(1.69)	(1.78)	(1.78)	(1.69)	(0.119)	
Empathy	3.88^b^	4.02^a^	3.95^1^	3.83^b, 2^	10.12	< 0.001
	(0.58)	(0.55)	(0.62)	(0.64)	(0.119)	
Maladjustment
Aggression	1.92^a^	1.83^b, 1^	1.76^b^	1.74^b, 2^	14.75	< 0.001
	(0.50)	(0.49)	(0.47)	(0.49)	(0.144)	
Hostile sexism	2.24^b^	2.10^b, 2^	2.36^b, 1^	2.84^a^	32.44	< 0.001
	(1.08)	(1.09)	(1.14)	(1.23)	(0.214)	

Bonferroni test *α* = 0.05; a > b; 1 > 2.

Examining the age profiles for the maladjustment indicators (see Figure 1), aggression showed a declining tendency from adolescence to later life. The lowest aggression levels corresponded to older adults, who scored lower than young adults and adolescents. By contrast, adolescents scored higher than young, middle-aged, and older adults. Hostile sexism showed an increasing tendency from young adulthood to later life. Older adults scored higher than middle-aged, young adults, and adolescents. Furthermore, young adults (who did not differ from adolescents) scored lower than middle-aged adults.

Additionally, when analyzing sex differences (see Table 4), on the adjustment indicators, males scored higher on self-esteem and emotional self-concept than females, but lower on empathy. On the maladjustment indicators, the lowest aggression corresponded to females, and on hostile sexism, males again scored higher than females.

**Table 4:** Means and (standard deviations) for sex, univariate *F*-values, and (*f* Cohen) for adjustment (self-esteem, emotional self-concept, and empathy) and maladjustment (aggression and hostile sexism) criteria.

	Sex		*F*(1, 2,126)	Value of *p*
	Female	Male		
Adjustment
Self-esteem	2.55	2.58	13.43	<0.001
	(0.20)	(0.19)	(0.079)	
Emotional self-concept	5.27	6.18	147.02	<0.001
	(1.72)	(1.66)	(0.263)	
Empathy	4.03	3.77	106.90	<0.001
	(0.57)	(0.60)	(0.224)	
Maladjustment
Aggression	1.79	1.87	15.24	<0.001
	(0.47)	(0.52)	(0.085)	
Hostile sexism	2.09	2.69	134.12	<0.001
	(1.04)	(1.22)	(0.251)	

Bonferroni test *α* = 0.05.

### 3.4. The early experiences in the family

The results of the univariate analyses showed statistically significant differences between the parenting styles on all the adjustment and maladjustment criteria, *p* < 0.05 (see Table 5). On the adjustment criteria, in adolescents and adult children, the only parenting style constantly related to the most positive scores was indulgent parenting. On self-esteem, children from authoritarian families scored lower than their peers from the other households. On emotional self-concept, children with indulgent parents obtained the highest scores, whereas authoritative, authoritarian, and neglectful parenting were related to a poor emotional self-concept. On empathy, children raised in authoritative and indulgent families scored higher than their counterparts from authoritarian and neglectful homes, whereas of the parenting styles related to poor empathy (i.e., authoritarian and neglectful), the lowest scores corresponded to neglectful parenting.

**Table 5:** Means and (standard deviations) of parenting style, univariate *F*-values, and (*f* Cohen) for adjustment (self-esteem, emotional self-concept, and empathy) and maladjustment (aggression and hostile sexism) criteria.

	Parenting style				*F*(3, 2,126)	Value of *p*
	Indulgent	Authoritative	Authoritarian	Neglectful		
Adjustment
Self-esteem	2.58^a^	2.58^a^	2.52^b^	2.56^a^	9.50	<0.001
	(0.19)	(0.20)	(0.20)	(0.19)	(0.116)	
Emotional self-concept	5.93^a^	5.59^b^	5.48^b^	5.55^b^	9.10	<0.001
	(1.78)	(1.80)	(1.70)	(1.70)	(0.113)	
Empathy	4.12^a^	4.05^a^	3.80^b^	3.71^c^	67.41	<0.001
	(0.49)	(0.51)	(0.61)	(0.65)	(0.308)	
Maladjustment
Aggression	1.67^c^	1.78^b^	1.92^a^	1.93^a^	36.98	<0.001
	(0.41)	(0.47)	(0.52)	(0.51)	(0.228)	
Hostile sexism	2.15^b^	2.36^a^	2.46^a^	2.40^a^	9.90	<0.001
	(1.10)	(1.15)	(1.21)	(1.13)	(0.118)	

Bonferroni test *α* = 0.05; a > b > c.

A similar pattern was found for the maladjustment criteria in adolescents and adult children. Indulgent parenting was the only parenting style constantly associated with the lowest scores. On aggression, children from indulgent homes obtained the lowest scores, and the highest scores corresponded to their peers from authoritarian and neglectful homes, whereas children from authoritative homes scored in a middle position between the most aggressive (i.e., from authoritarian and neglectful families) and the least aggressive (i.e., from indulgent homes). On hostile sexism, children with indulgent parents obtained the lowest scores, compared to children raised by authoritative, authoritarian, and neglectful families.

**Figure 2:**
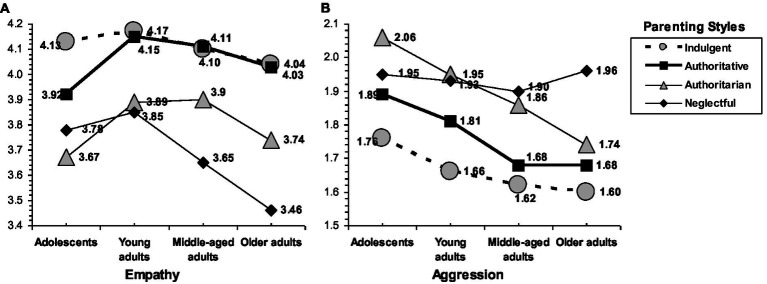
Interaction of parenting styles by age on empathy **(A)** and aggression **(B)**.

Statistically significant effects of parenting style and age were found for empathy, *F*(9, 2,126) = 3.569, *p* < 0.001, and aggression, *F*(9, 2,126) = 1.955, *p* = 0.041. Examining the family profiles for empathy by age (see Figure 2), a common pattern can be observed in adolescents and adult children: indulgent and authoritative parenting were related to high scores (in adolescence the greatest empathy corresponded to the indulgent style), whereas authoritarian and neglectful parenting were associated with low empathy (in middle and later life, the lowest scores corresponded to the neglectful style). Additionally, empathy declined with age, but not in all families. In families characterized by warmth, adult children tended to preserve their empathy: older adults scored similar to young adults from indulgent and authoritative families. By contrast, a decline in empathy was found in families without warmth: older adults reported lower scores than young adults from authoritarian and, remarkably, neglectful homes.

Examining the family profiles for aggression by age (see Figure 2), a decreasing tendency was found over time in children raised by authoritative, indulgent, and authoritarian parents. The neglectful parenting style did not present this decreasing pattern, given that it was related to high and constant levels of aggression from adolescence to later life. Additionally, authoritative and indulgent parenting were related to low aggression (the lowest scores corresponded to indulgent parenting in adolescence, adulthood, and later life). By contrast, children from authoritarian and neglectful families tended to report high aggression (the highest scores corresponded to authoritarian parenting in adolescence and to neglectful parenting in later life, whereas they were practically identical in young adulthood and middle life).

## 4. Discussion

This study analyzed age-related differences in adjustment (i.e., self-esteem, emotional self-concept, and empathy) and maladjustment (i.e., aggression and hostile sexism) across adolescence, adulthood, and later life. Additionally, we tested whether differences in adjustment and maladjustment in adolescence, adulthood, and later life could be related to early experiences in the family (authoritative, indulgent, authoritarian, and neglectful). Different age-related differences in adjustment (i.e., self-esteem, emotional self-concept, and empathy) and maladjustment (i.e., aggression and hostile sexism) were found. Later life was related to some negative scores (lower self-esteem and empathy and higher hostile sexism), but the opposite pattern for positive scores was found in other dimensions (higher emotional self-concept and lower aggression). Additionally, early experiences in the family were related to adjustment and maladjustment in adolescent and adult children. Overall, only parenting characterized by warmth without strictness (the indulgent style) was constantly related to optimal scores.

Different age-related differences in adjustment and maladjustment to later life were identified. On the adjustment criteria, age profiles in self-esteem showed an increasing tendency from adolescence, reaching a peak in middle-adulthood, and decreasing in later life. Surprisingly, a different pattern was found when the self was examined based on a multidimensional approach (i.e., self-concept). Specifically, for emotional self-concept, there was an age-related increasing tendency throughout life. Older adults had the greatest emotional self-concept (they did not differ from middle-aged adults), scoring higher than young adults and adolescents. Findings from this study agree with age-related differences in global self-perceptions (i.e., self-esteem) described in some previous studies (e.g., Liu et al., 2019), but they also provide new evidence because the present study examined the self from a multidimensional perspective (i.e., self-concept). Based on a unidimensional perspective, a dramatic decline in self-worth from middle to later life was previously described. However, at the same time, older adults seem to preserve greater self-perceptions in important domains of the self, such as the emotional domain, perhaps partly because emotion regulation is relatively spared (Carstensen and Mikels, 2005). Finally, on empathy, older adults reported low scores, agreeing with findings from some previous studies limited to comparisons between older and young adults (e.g., Bailey et al., 2008). Findings from the present study offer new and crucial evidence about age-related differences in empathy throughout life. Older adults and adolescents had the lowest empathy (they did not differ from each other), whereas the highest levels were found among young adults. In addition, middle-aged adults reported higher empathy than older adults.

On the maladjustment criteria, age profiles for aggression showed a decreasing tendency from adolescence to later life. The highest aggression corresponded to adolescents, who scored higher than young, middle-aged, and older adults. By contrast, the lowest levels of aggression corresponded to older adults, who reported lower scores than young adults and adolescents. The opposite was true for hostile sexism. The highest hostile sexism corresponded to older adults, who scored higher than young adults, middle-aged adults, and adolescents. By contrast, the lowest levels of hostile sexism were found in young adults, who did not differ from adolescents but scored lower than middle-aged and older adults. Overall, previous studies showed that higher prejudice (e.g., sexism and racism) was related to greater aggression (Genthner and Taylor, 1973; Lisco et al., 2012). However, most of these studies only examined the relationship between prejudice and aggression in a specific age group (i.e., young adults), without considering the age-related differences throughout life, which offers a more general profile and might even disagree with age-related partial correlates (Genthner and Taylor, 1973; Lisco et al., 2012). Interestingly, the main findings from the present study revealed different age-related differences in sexism prejudice and aggression: later life is related to high hostile sexism, but low aggression, whereas adolescence and young adulthood, although related to less hostile sexism, are associated with high aggression.

An important aim of the present study was to analyze age differences in three indicators of adjustment and two indicators of maladjustment. As expected, in later life, older adults had relatively low self-esteem (Orth et al., 2018). However, their capacity for emotional regulation appears to be good, despite declining physical capacities. Thus, both the high emotional self-concept and the low aggression could be explained in part by the fact that in later life emotional regulation might be high due to accumulated experiences (Carstensen and Mikels, 2005). A more comprehensive and complete understanding of differences across the life span requires a complete portrait with indicators of adjustment and maladjustment. Thus, older adults also showed high scores on hostile sexism and low scores on empathy, although their aggression was also low. The multidimensional study across four age groups has important implications. Although greater vulnerability (i.e., low scores on adjustment or high scores on maladjustment) is associated with more risk and problems, identifying profiles by age in different dimensions can provide a more general view of those protective and risk factors. For example, according to the findings of the present study, in later life, there is low confidence in one’s abilities (low self-esteem), which may increase the risk of depression and loneliness (Bergman and Bodner, 2020), although it does not necessarily mean that all areas of the self are affected. For example, appraising oneself as being capable and effective in coping with the situation by controlling one’s emotions (high emotional self-concept) is also a protective factor against mental health problems.

Another relevant aim of the study was to test whether, despite age differences in adjustment and maladjustment, early experiences in the family could also be related to consistent variations in adjustment and maladjustment. The main findings showed that early family experiences (authoritative, authoritarian, indulgent, and neglectful) were related to important differences in adjustment and maladjustment. In general, and as expected, only indulgent parenting (warmth but not strictness) was associated with the most positive scores. It should be noted that there are many influences on adjustment and maladjustment, especially from adolescence to later life (Baltes, 1987), and the family has been consistently identified as a protective factor, but also as a risk factor.

The findings from this study suggest that early experiences in the family are crucial in explaining differences in adjustment and maladjustment in adolescents, but also in adult children. Overall, on the adjustment criteria, the only parenting style consistently related to the most positive scores was the indulgent style. Specifically, children from indulgent families scored equally on self-esteem and empathy and better on emotional self-concept than those from authoritative homes. By contrast, parenting without warmth (i.e., authoritarian and neglectful) was related to low scores on emotional self-concept and empathy (children from authoritarian homes reported the lowest scores on self-esteem). Again, a similar pattern was found for maladjustment: Indulgent parenting was the only parenting style constantly associated with the lowest scores. Specifically, indulgent parenting was associated with lower aggression and less hostile sexism, compared to authoritative, authoritarian, and neglectful parenting. Additionally, children from authoritarian and neglectful families were the most aggressive.

The present findings seriously contradict those from classic studies with European-American families. According to these studies, parental warmth combined with parental strictness (i.e., authoritative parenting) is the only style that helps children to achieve the greatest adjustment and the lowest maladjustment (Maccoby and Martin, 1983; Darling and Steinberg, 1993; Steinberg et al., 1994). The strictness component is identified as effective and necessary in European-American families to achieve effective socialization based on good internalization of social standards and self-regulation (Baumrind, 1967; Baumrind, 1971; Steinberg, 2001). However, according to the main findings of the present study, parenting based on involvement and warmth might be consistently related to benefits, whereas parental strictness seems to be unnecessary and even harmful. Within parenting characterized by warmth, indulgent parenting (without warmth) obtains scores equal to those of the authoritative style (without warmth) on self-esteem and empathy, and more optimal scores on emotional self-concept, aggression, and hostile sexism. The strictness component, although combined with parental warmth, does not always seem to provide a good social standard because adolescent and adult children raised in authoritative families reported greater hostile sexism, had seriously damaged emotional regulation because they tended to report lower emotional self-concept, and were very aggressive, which implies a serious alteration in personal regulation and lack of self-control, making it difficult to live in society. In contrast, when parents have been loving and involved and have used reasoning and dialog at home, without making use of imposition and severity (i.e., the indulgent families), their adolescent and adult children tend to be more empathetic, more appreciative of themselves and their emotional abilities, and better adjusted because they are less prejudiced and less aggressive. In this regard, the findings from the present study agree with some recent research, mostly conducted in Europe and Latin America, that identifies the benefits of indulgent parenting based on warmth without strictness (Calafat et al., 2014; Garcia et al., 2019; Martinez et al., 2020). Most of these previous studies are limited to samples of adolescents (Queiroz et al., 2020), and so the present study extends the evidence about the benefits of indulgent parenting to adulthood and later life.

Overall, parenting styles and adjustment and maladjustment in adolescent and adult children showed a common pattern, but the parenting profiles by age revealed some nuances in empathy and aggressiveness. Examining the age profile, a decreasing tendency in empathy was found from young adulthood to later life. Older adults scored lower than middle-aged and young adults. Nevertheless, when examining family age profiles, children from families characterized by warmth (i.e., authoritative and indulgent) did not have this decreasing tendency (older adults scored similarly to middle-aged and young adults). Thus, parental warmth could have a buffering effect on the empathy decline from young adulthood to later life, whereas lower scores on empathy in older adults than in young adults were found in families characterized by lack of warmth (remarkably in those from neglectful homes). Additionally, aggression tended to decrease with age. Older adults were less aggressive than young adults and adolescents. However, examining family age profiles of children raised in neglectful families, scores were as high in adolescence as in later life. By contrast, a common pattern across adolescence, adulthood, and later life was found: Indulgent parenting was related to the lowest levels of aggression.

### 4.1. Limitations

The present study offers new and crucial evidence, but some limitations should be considered. We cannot determine a relationship of causality between the variables because the methodology is not experimental and the design is cross-sectional rather than longitudinal. This study has not been designed to be representative of the Spanish population, although other representative Spanish data offer similar results (Alonso-Geta, 2012). The results of this study are from middle-class families, and so future studies should examine parental socialization in families from other environments. Nevertheless, the results of the present study are similar to those obtained in other studies with Spanish families from at-risk neighborhoods (Gracia et al., 2012; Fuentes et al., 2015a). It is important to bear in mind that parenting is measured based on the two main dimensions that make up the four parenting styles (i.e., authoritative, authoritarian, indulgent, and neglectful), providing a family categorization that is different from other parenting studies in which parental socialization is captured through a clustering method such as LPA (i.e., latent profile analysis; Power, 2013; Borden et al., 2014; Ayon et al., 2015; Huang et al., 2022). Additionally, adjustment and maladjustment have been measured with self-report measures, so future studies might also include more tangible measures. Despite the use of self-report measures to capture parenting and adjustment and maladjustment, children seem to offer more reliable and accurate data than other sources (e.g., parents; Gonzales et al., 1996; Barry et al., 2008). Moreover, the sample used in this study includes almost the entire life cycle, which makes it possible to examine differences in adjustment and maladjustment criteria throughout life, as well as the impact of early experiences (i.e., parenting style) in the short and long term.

## Data availability statement

The raw data supporting the conclusions of this article will be made available by the authors via email request to the corresponding author, without any undue reservation.

## Ethics statement

The studies involving human participants were reviewed and approved by the College Research Ethics Committee (CREC) of Nottingham Trent University (protocol code no. 2017/90, May 2017). Written informed consent to participate in this study was provided by the participants’ legal guardian/next of kin.

## Author contributions

MA, OG, PQ, and FG contributed to the conception and design of the study. MA and FG organized the database. MA and PQ performed the statistical analysis. MA and OG wrote the first draft of the manuscript. PQ and FG wrote sections of the manuscript. All authors contributed to the manuscript revision and read and approved the submitted version.

## Funding

The research reported in this study has been supported by grant CIAICO/2021/252 (Conselleria for Innovation, Universities, Science and Digital Society, Generalitat Valenciana), which provided the support for open-access publication fees. Additionally, it has been partially supported by grants FPU20/06307 (Ministry of Universities, Government of Spain), and ACIF/2016/431 and BEFPI/2017/058, which provided funding for a research stay at Nottingham Trent University, United Kingdom (Generalitat Valenciana and European Social Fund).

## Conflict of interest

The authors declare that the research was conducted in the absence of any commercial or financial relationships that could be construed as a potential conflict of interest.

## Publisher’s note

All claims expressed in this article are solely those of the authors and do not necessarily represent those of their affiliated organizations, or those of the publisher, the editors and the reviewers. Any product that may be evaluated in this article, or claim that may be made by its manufacturer, is not guaranteed or endorsed by the publisher.
